# Long-term survival of 11 years with multidisciplinary therapy for hepatocellular carcinoma metastasis to the ovary and peritoneum: a case report

**DOI:** 10.1007/s12328-021-01434-2

**Published:** 2021-05-12

**Authors:** Satoko Motegi, Takeshi Yokoo, Ryosuke Nozawa, Rie Azumi, Yuzo Kawata, Kohei Ogawa, Toru Setsu, Ken-ichi Mizuno, Koji Nishino, Hajime Umezu, Hirokazu Kawai, Takeshi Suda, Shuji Terai

**Affiliations:** 1 Division of Gastroenterology and Hepatology, Graduate School of Medical and Dental Sciences, Niigata University, Niigata, Niigata Japan; 2 Department of Preemptive Medicine for Digestive Diseases and Healthy Active Life, School of Medicine, Niigata University, Niigata, Niigata Japan; 3 Department of Obstetrics and Gynecology, Graduate School of Medical and Dental Sciences, Niigata University, Niigata, Niigata Japan; 4 Division of Pathology, Graduate School of Medical and Dental Sciences, Niigata University, Niigata, Niigata Japan; 5 Department of Internal Medicine, Niigata Prefectural Shibata Hospital, Shibata, Niigata Japan; 6 Department of Gastroenterology and Hepatology, Uonuma Institute of Community Medicine Niigata University Hospital, Minamiuonuma, Niigata Japan

**Keywords:** Hepatocellular carcinoma, Ovary, Peritoneum, Multidisciplinary therapy, Case report

## Abstract

We herein report a rare case of HCC metastases to the ovary and peritoneum in a 61-year-old female patient who has achieved 11-year survival with multidisciplinary therapy. The patient was diagnosed with HCC during balloon angioplasty performed for Budd–Chiari syndrome in 1994 and underwent partial hepatectomy twice. Five years after the second hepatectomy, allochronic recurrence of a single nodule detected in S8 was treated by radiofrequency ablation, followed by percutaneous ethanol injection therapy and stereotactic body radiotherapy. However, her α-fetoprotein level rose to 1862 ng/mL within one year and computed tomography revealed a large pelvic tumor suggesting HCC metastasis to the ovary. The subsequent laparotomy revealed one 11-cm left ovarian tumor, one small right ovarian nodule, and numerous peritoneal nodules. Bilateral salpingo-oophorectomy and peritoneal resection of as many nodules as possible were performed. Combination therapy with intravenous 5-fluorouracil plus cisplatin and ramucirumab monotherapy effectively suppressed tumor progression with maintenance of hepatic functional reserve, and she has achieved long-term survival of 11 years, illustrating that multidisciplinary therapy with favorable hepatic functional reserve maintenance can contribute to long-term survival in HCC with extrahepatic spread.

## Introduction

The lungs, lymph nodes, bones, and adrenal glands are the most common metastatic sites, and the reported median survival after the diagnosis of extrahepatic spread is 8.1 months [1]. Importantly, the prognosis remains poor in these patients despite the recently developed molecular-targeted agents. We herein report the case of a patient with rare ovarian and peritoneal metastases of HCC who achieved 11-year survival by longitudinal multidisciplinary therapy, which afforded effective maintenance of hepatic functional reserve.

## Case report

**Fig. 1:**
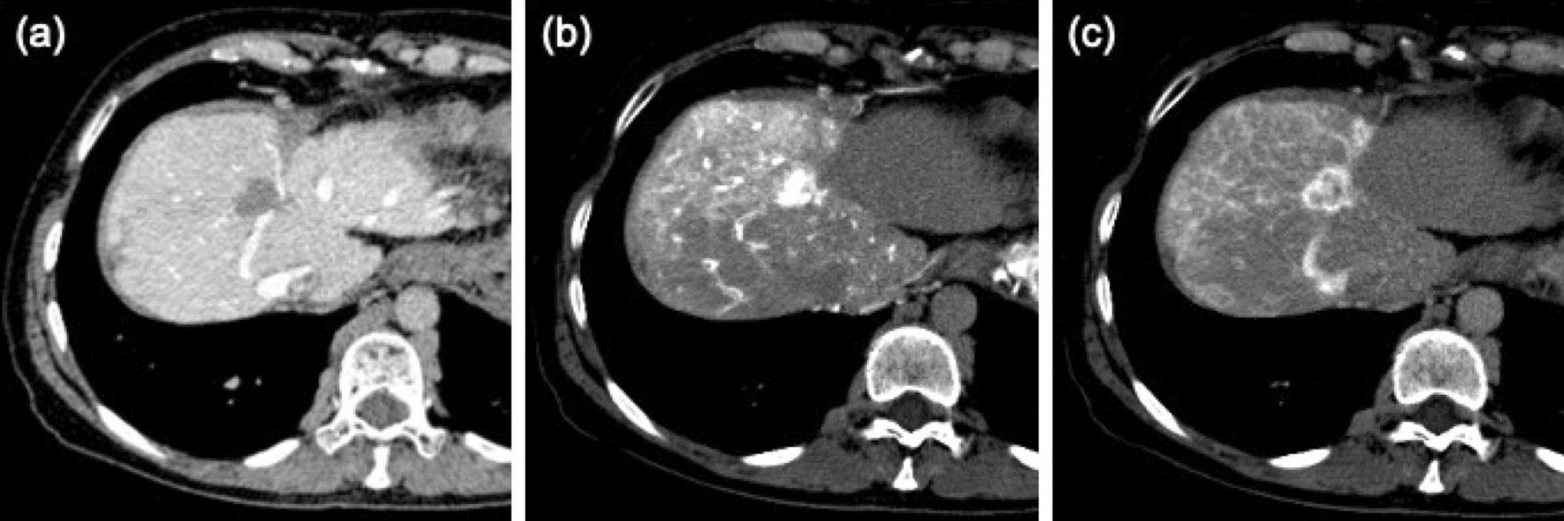
Computed tomography images during angiography at the time of S8 nodule diagnosis in 2007. **a** Computed tomography (CT) during arterioportography image showing a defect in the portal supply to the tumor. **b**, **c** CT during right hepatic arteriography images showing overt enhancement followed by corona-like enhancement, a typical presentation of hepatocellular carcinoma

A 61-year-old female patient was diagnosed with liver cirrhosis due to Budd–Chiari syndrome and underwent balloon angioplasty in 1994. At the time, she was also diagnosed with HCC located in S7 and underwent partial hepatectomy one month later. Eight years after the initial HCC diagnosis, two new HCC nodules found in S2 and S3 were removed by partial hepatectomy as curative surgery. Five years after the second hepatectomy, she experienced another HCC recurrence in S8, which presented as corona-like enhancement in computed tomography (CT) images during hepatic arteriography (Fig. 1). Although sequential treatment with radiofrequency ablation (RFA) and percutaneous ethanol injection therapy (PEIT) was performed, local recurrence was detected seven months after the treatment. The patient underwent RFA again. As a result, a liver abscess was developed at the ablation site. After the resolution of abscess with antibiotic treatment, remnant HCC was visualized. Stereotactic body radiotherapy was performed as additional treatment eleven months after the second RFA.

However, her α-fetoprotein (AFP) level reached 1862 ng/mL ten months after stereotactic body radiotherapy (about 2.5 years later from diagnosis of S8 nodule). Neither CT nor magnetic resonance imaging (MRI) showed HCC recurrence in the liver or the upper abdomen, but gynecological examination performed to diagnose lower abdominal discomfort led to the identification of a non-tender, hard mass in the lower abdomen.

**Fig. 2:**
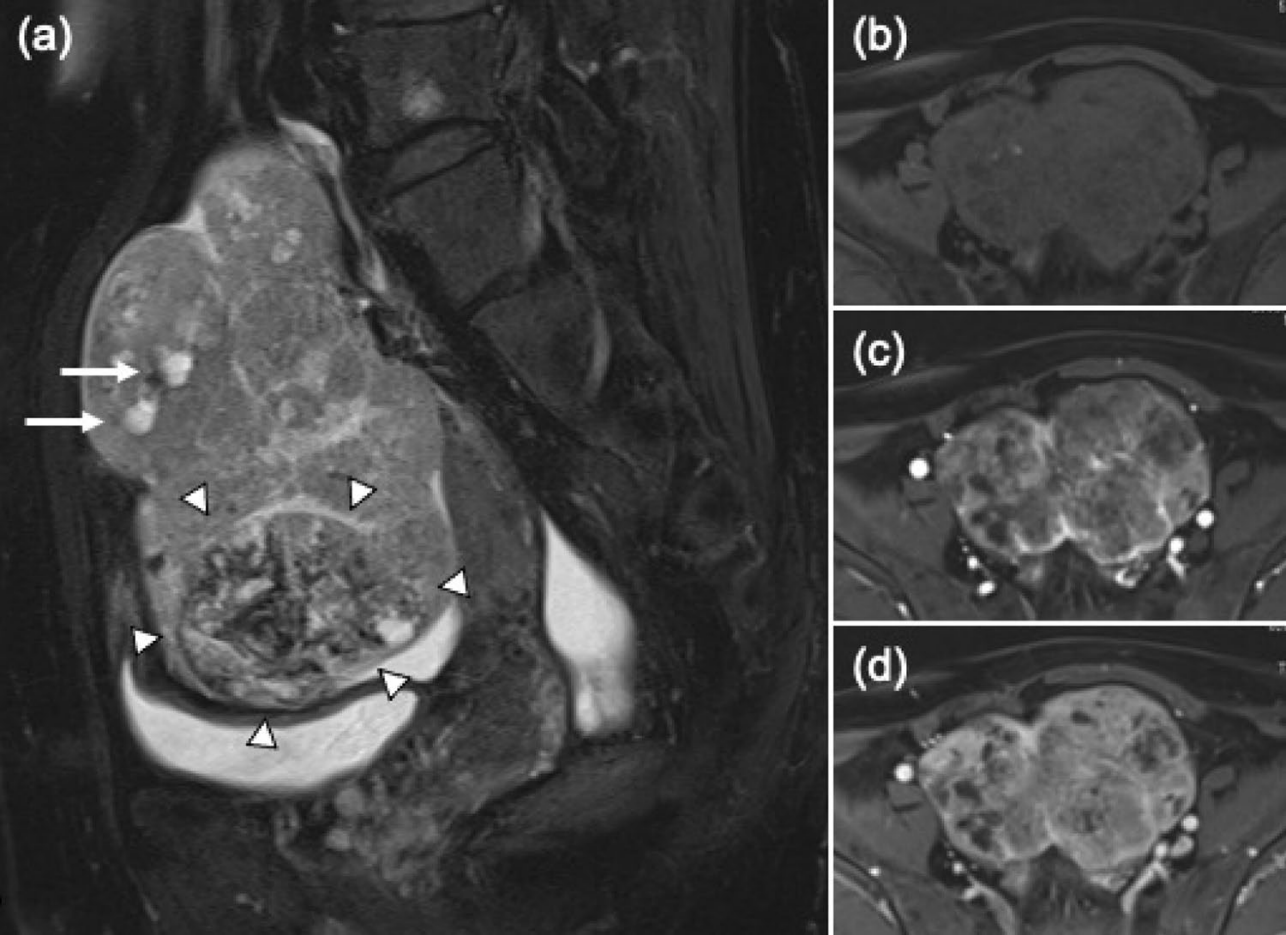
Magnetic resonance images of pelvis. **a** T2-weighted sagittal image showing a 11-cm tumor including cystic (arrows) and necrotic lesions with hemorrhage (arrowheads). **b**–**d** Dynamic contrast-enhanced magnetic resonance images showing tumor hypervascularity (**b** plain phase; **c** early phase; **d** delayed phase)

The results of laboratory tests performed at the time are presented in Table 1. The levels of AFP and des-γ-carboxy prothrombin (DCP) were elevated (2156 ng/mL and 2302 mAU/mL, respectively), whereas CA125 was slightly above the normal limit (123 U/mL). Her hepatic functional reserve was Child–Pugh A (6 points), and she was in modified albumin-bilirubin (mALBI) grade 2a (score, − 2.45). Her renal function was not impaired. MRI indicated a large tumor measuring 11 × 10 × 6 cm in the pelvic cavity (Fig. 2a–d), and the ovary was considered as the origin of the tumor, which was hypervascular and included necrotic areas. No HCC recurrence was detected in the liver by dynamic CT and MRI. No tumor was observed in lymph nodes, lungs, bones, and adrenal glands. Endoscopy did not show any tumors in esophagus, stomach, duodenum, and colon. The pelvic tumor was strongly suspected to be an HCC metastasis based on the patient’s medical history, high levels of AFP and DCP, and no evidence of tumors in other organs.

**Table 1:** Laboratory findings at the time of ovarian tumor diagnosis

TP	7.9	g/dL	AFP	2156	ng/mL
Alb	3.8	g/dL	AFP-L1	95.3	%
T-Bil	0.9	mg/dL	AFP-L3	4.7	%
AST	50	U/L	DCP	2302	mAU/mL
ALT	22	U/L	CA125	123	U/mL
BUN	20	mg/dL	CEA	5.1	ng/mL
Cre	0.54	mg/dL	CA19-9	17	U/mL
NH3	113	μg/dL			
PLT	19.6	× 10^4^/uL	Encephalopathy		None
PT	85	%	Ascites		Mild
PT-INR	1.09				
			Child–Pugh		A (6)
			mALBI grade		2a (− 2.45)
			FIB-4 index		3.32

Bilateral salpingo-oophorectomy as curative therapy was attempted in December 2009. However, in addition to the large ovarian tumor, numerous small nodules in the peritoneum and mesentery were detected during laparotomy, and as many disseminated nodules as possible were resected in addition to bilateral salpingo-oophorectomy.

**Fig. 3:**
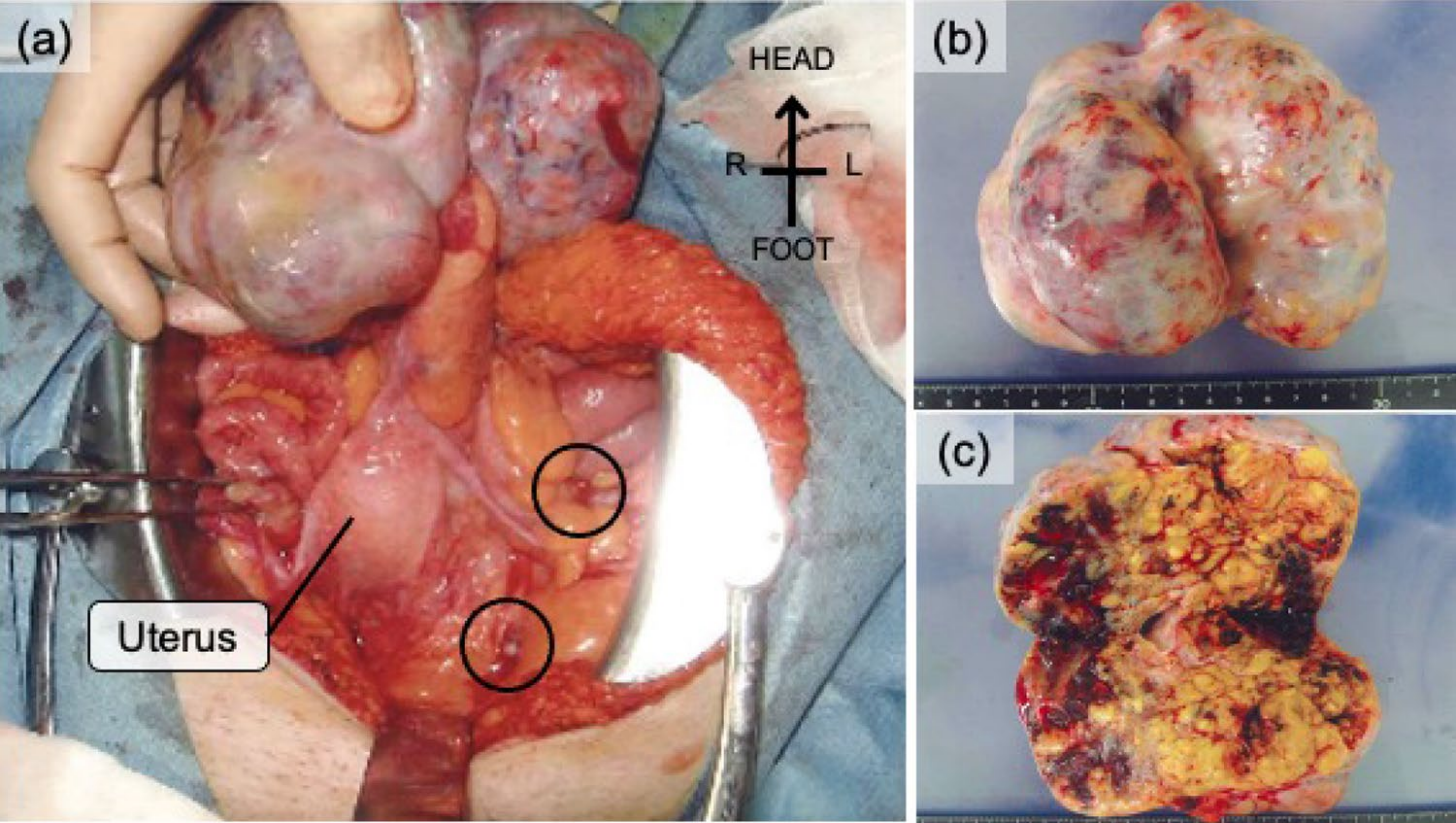
Gross findings of the pelvic cavity during laparotomy. **a** Findings in the pelvic cavity. Left ovary is the origin of the large tumor. Black circles indicate peritoneal nodules. **b** Gross view of the resected tumor originating from the left ovary. The tumor dimensions are 11 × 10 × 6 cm. **c** The internal structure is yellowish and solid, and partial intertumoral hemorrhage is visible

**Fig. 4:**
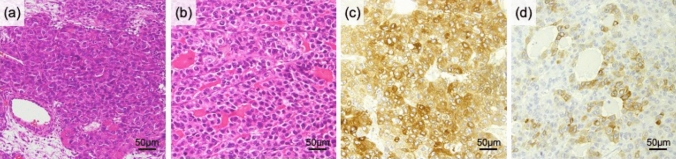
Representative microscopic images of the left ovarian tumor. **a**, **b** Hematoxylin–eosin staining. Eosinophilic cells with high nuclear/cytoplasm ratio propagate in a compact pattern. **c** Cells positive for Hep-Par1 are heterogeneously detected in the tumor. **d** CK7-positive cells forming glandular structures are partly observed in the tumor

Pathological evaluation revealed a yellow solid tumor in the left ovary, 11 × 10 × 6 cm in size, with hemorrhagic and cystic components, partially with aggregations of small nodules (Fig. 3). A nodule, 1.3 × 0.9 cm in size, was detected in the right ovary. Microscopic evaluation showed similar findings in both ovarian tumors, which exhibited alveolar, trabecular, and tubular patterns and comprised eosinophilic cells with a high nuclear/cytoplasm ratio. Immunohistochemical examination showed that the tumors were extensively positive for CAM5.2, partially positive for Hep-Par1 (Fig. 4c) and CK7 (Fig. 4d), and negative for CK20 and AE1/AE3 (data not shown). Peritoneal nodules, ranging from 0.2 to 1 cm in size, exhibited findings similar to those of the ovarian tumors. These findings were compatible with HCC. Based on the medical history and pathological findings, the ovarian and peritoneal tumors were diagnosed as HCC metastases. As a result of the surgery, remnant lesions were located only in the peritoneum because peritoneal resection could not remove all metastatic nodules. AFP decreased to 136 ng/mL.

**Fig. 5:**
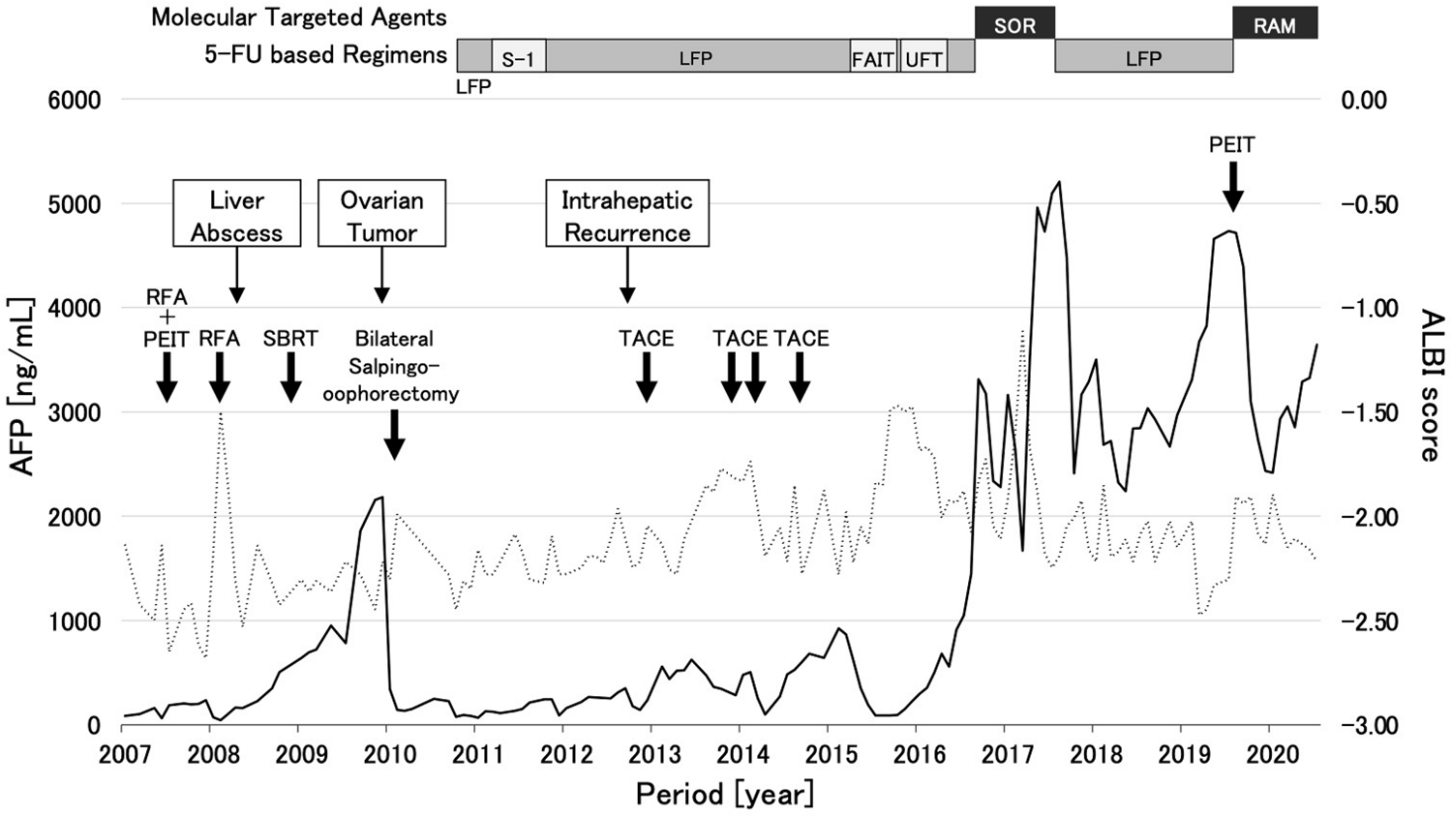
Clinical course during multidisciplinary therapy. Solid and dotted lines show changes in serum alpha-fetoprotein (AFP) levels (ng/mL) and modified albumin-bilirubin score, respectively, during the course of multidisciplinary therapy. *5-FU* 5-fluorouracil, *LFP* intravenous low-dose 5-FU and cisplatin, *S-1* tegafur/gimeracil/oteracil, *FAIT* Fluorouracil arterial infusion and interferon therapy, *UFT* tegafur/uracil, *SOR* sorafenib, *RAM* ramucirumab, *RFA* radiofrequency ablation, *PEIT* percutaneous ethanol injection therapy, *SBRT* stereotactic body radiation therapy, *TACE* transarterial chemoembolization

**Fig. 6:**
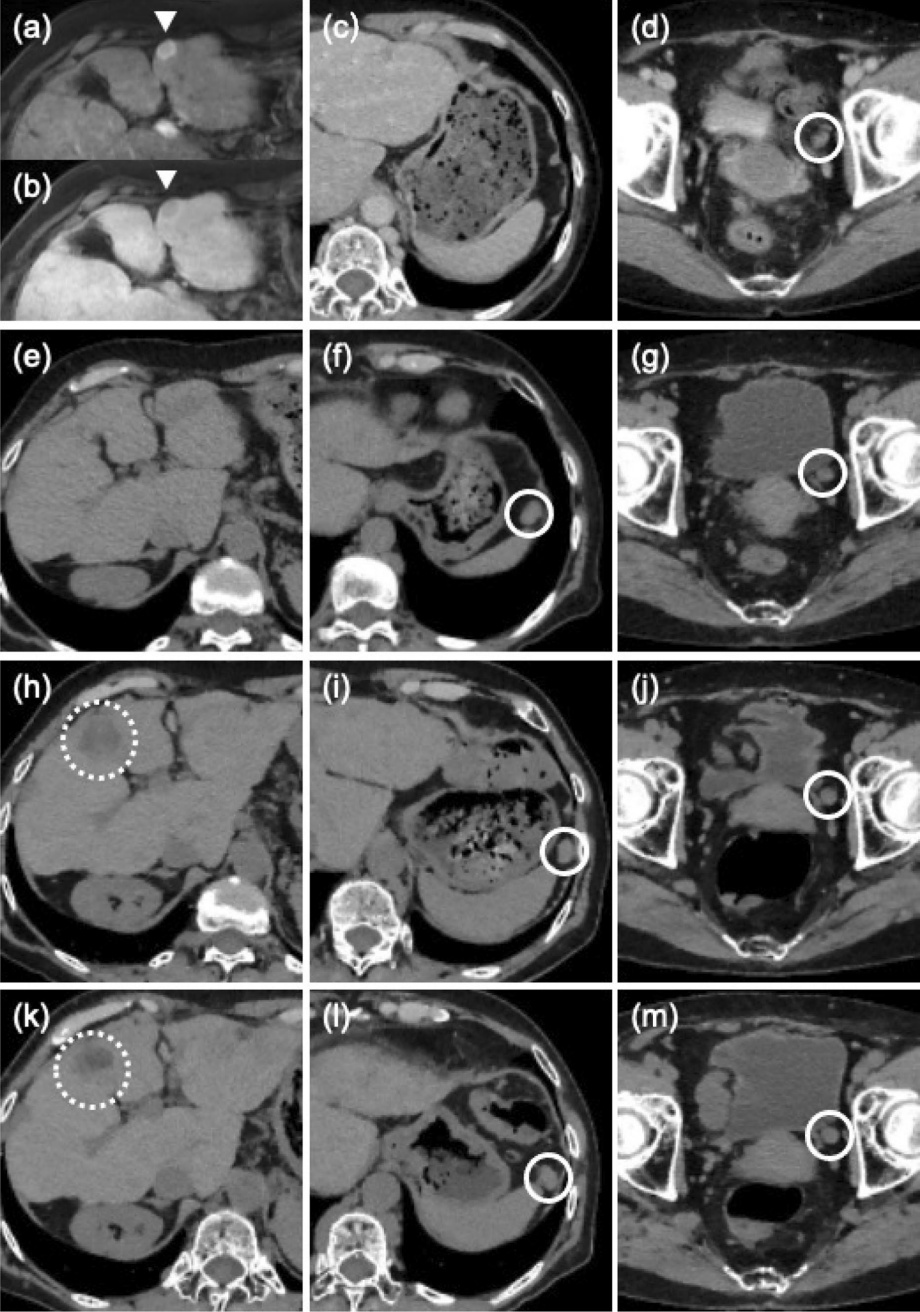
Representative images of the liver and abdomen after bilateral salpingo-oophorectomy. Intrahepatic recurrence was detected in 2012. Dynamic MRI showed early enhancement and wash out in early and delayed phase (arrow heads in **a**, **b**). Abdominal dissemination was indicated in the pelvis (**d**) and no nodule was near the spleen (**c**). Before administration of low-dose sorafenib in 2016 (**e**, **f**, **g**), a new disseminated lesion appeared near the spleen. Use of the contrast medium was avoided due to allergic reaction. Before percutaneous ethanol injection therapy (PEIT) in 2019, another intrahepatic lesion in S5 was clarified even in plain CT (**h**). The lesions of the abdominal dissemination were stable (**i**, **j**). After PEIT in 2020, The intrahepatic lesion in S5 include low density area which allowed to suggest necrosis (**k**). The lesions of the abdominal dissemination were stable (**l**, **m**). White solid and dotted circles show lesions of abdominal dissemination and an intrahepatic lesion in S5

Two months after bilateral salpingo-oophorectomy, sorafenib (600 mg/day) was initiated as systemic therapy but was withdrawn after 12 days because of severe hand-foot syndrome, which was equivalent to grade 3 in common terminology criteria for adverse events version 5.0. Starting in September 2010, 5-fluorouracil-based regimens, such as an intravenous 5-fluorouracil plus cisplatin (low-dose FP; 5-fluorouracil, 250 mg/body, days 1–5, 8–12, and 15–19 and cisplatin, 10 mg/body, days 1–5, 8–12, and 15–19), and tegafur/gimeracil/oteracil (S-1, 60 mg/day) monotherapy were administered. Although the regimens suppressed an increase of AFP level under 250 ng/mL for a year, a new intrahepatic lesion was detected in September 2012. On-demand transarterial chemoembolization (TACE) was added as locoregional therapy. In March 2015, the patient developed anaphylactic reaction to iodine-containing contrast medium, leading to the contraindication of TACE and contrast-enhanced CT; therefore, the patient was treated with 5-fluorouracil-based regimens, such as intravenous 5-fluorouracil plus interferon α-2a, and tegafur/uracil monotherapy. However, long-term tumor suppression was not possible, and transient reduction in hepatic functional reserve was observed. Renal function also started to decline from this period. Because estimated glomerular filtration rate decreased to 56.05 mL/min/1.73m^2^, low-dose sorafenib (200 mg/day) was administered as an alternative treatment and the severity of adverse effects, such as hand-foot syndrome and anorexia, were grade 2 and tolerable. However, the AFP level increased to 5096 ng/mL. Lenvatinib (4 mg), which was initiated as the next treatment, had to be discontinued at day 11 due to a reduction in estimated glomerular filtration rate from 41.01 to 29.31 mL/min/1.73m^2^. After the discontinuation, renal function returned to the former level. The subsequent re-initiation of intravenous low-dose FP with 30% dose of cisplatin could suppress tumor progression until 2018, as shown in Figs. 5 and 6.

In 2019, four hepatic lesions were newly detected by plain CT and contrast-enhanced ultrasound. Percutaneous ethanol injection therapy for the intrahepatic nodule in S5, which grew more rapidly compared to the other three nodules, was followed by ramucirumab monotherapy. Although the treatment was briefly interrupted due to ophthalmic surgery and proteinuria, her AFP level transiently decreased from 4717 to 2418 ng/mL. With the sequential treatment, the patient has been able to maintain improved quality of life and good performance status for 11 years after the initial diagnosis of HCC metastases to the ovary and peritoneum. Because her mALBI score has exhibited only a slight increase from − 2.45 to − 2.30 despite the long-term treatment, multidisciplinary treatment has been ongoing.

## Discussion

Ovarian involvement is extremely rare in HCC. To date, only 16 living cases of HCC metastasis to the ovary have been reported except the present case (Table 2) [2–15]. The first two cases were reported in 1983 by Nakashima et al*.*, who reported one case (0.4%) in an autopsy cohort of 232 HCC cases [16], and by Oortman et al*.*, who reported a living patient [2]. The median age of these reported cases is 44 (range 27–76) years. Unilateral and bilateral involvement was observed in 9 and 8 patients, respectively, and 8 of the 17 cases had peritoneal metastasis.

**Table 2:** Summary of 16 reported cases of HCC metastasis to ovaries

Case	Age	Previous treatment			Tumor characteristics		Location of viable lesion at surgery			Treatment			Report	
		Hepatic surgery	Puncture or rupture	Others	Maximum size of ovary [cm]	AFP [ng/ml]	Liver	Other organs	Ovarian site	Salpingo-oophorectomy	Other treatment	Prognosis after SO [months]	Year	Ref
1	36	Hepatectomy	Rupture	–	6	18,800	–	–	Left	Bilateral SO (with TAH)	–	N/A	1983	2
2	38	Hepatectomy	–	–	4–11**	14,819	–	–	Left	Bilateral SO (with TAH)	Cx*	~ 52	1992	4
3	47	Liver transplantation	–	–	20	4	–	–	Right	Bilateral SO	Radiation (to bone meta)	12	2000	5
4	43	Hepatectomy	RFA	TACE	7	336,520	–	–	Right	Right SO	–	N/A	2011	6
5	44	–	RFA	–	7	2.8	–	–	Right	Bilateral SO	–	~ 54 alive cancer free	2017	3
6	44	Liver transplantation	–	–	11	58	–	–	Bilateral	Bilateral SO	(sirolimus)	14 alive cancer free	2005	8
7	27	Hepatectomy	RFA	TACE	3.2	N/A	–	–	Bilateral	Bilateral SO	–	N/A	2017	7
8	31	–	–	–	4–11**	2700	Multiple*	–	Bilateral	Bilateral SO	Cx*	18	1992	4
9	66	–	Biopsy	–	17	55	Multiple	–	Bilateral	Bilateral SO	–	6	1994	9
10	61	–	PEIT	–	N/A	350,000	N/A	–	Right	Bilateral SO (with TAH)	N/A	N/A	2001	10
11	39	–	–	TACE, Cx*	N/A	60,000 <	–	Lung, spleen, lymph node	Right	N/A	Cx*	N/A	2004	11 (Korean)
12	68	–	–	–	4–11**	11,000	single	Peritoneum, lymph node	Bilateral	Bilateral SO, omentectomy	Cisplatin	5 alive	1992	4
13	76	Hepatectomy with ileocecal resection due to adhesion	–	TACE, SOR	~ 2.5	–	Single	Peritoneum	Left	Left SO, hepatectomy	SOR cisplatin (to lung meta)	~ 32 alive cancer free	2013	12 (Japanese)
14	43	–	–	–	6.5	140	Multiple	Peritoneum	Left	Bilateral SO*	TAE, Cx*	5 alive	2005	13
15	56	–	–	–	15	534	Multiple	Peritoneum	Bilateral	Bilateral SO (with TAH and sigmoidectomy)	–	4	1999	14
16	40		RFA	TACE	N/A	2150	N/A	Peritoneum	Bilateral	N/A	–	N/A	2006	15 (Korean)
17	61	Hepatectomy	RFA, PEIT	TACE, SBRT	11	1862	–	Peritoneum	Bilateral	Bilateral SO, peritoneal resection	5-FU based regimens, SOR, ramcirumab	132 alive	Present case	

*RFA* radiofrequency ablation, *PEIT* percutaneous ethanol injection therapy: TA(C)E, transarterial (chemo)embolization, *Cx* chemotherapy, *SOR* sorafenib, *SBRT* stereotactic body radiation therapy, *SO* salpingo-oophorectomy, *TAH* total abdominal hysterectomy, *5-FU* 5-fluorouracil, *N/A* not applicable

^*^Details not known

**The sizes of five ovaries with tumors in these three cases were 4, 5, 6.5, 10, and 11 cm. Individual sizes are not described

In patients with HCC metastasis to the ovary, hepatoid yolk sac tumor and primary hepatoid carcinoma of the ovary, both AFP-producing ovarian tumors, should be considered in differential diagnosis. Hepatoid yolk sac tumor is commonly reported in young female patients, with rare occurrence reported in postmenopausal women [17]. Hepatoid carcinoma of the ovary is an uncommon ovarian tumor, first reported in 1987 [18]. The morphological and immunohistochemical features of hepatoid carcinoma of the ovary resemble those of HCC [19–21]. In the present case, the pathological findings were compatible to HCC. Furthermore, the postmenopausal age, history of radiofrequency ablation therapy and percutaneous ethanol injection therapy which can cause ovarian and peritoneal involvement [3, 22], and no evidence of tumors in other organs strongly supported the diagnosis of HCC metastasis to the ovary.

Prognosis of HCC with extrahepatic spread is poor. A meta-analysis reported that 1-year survival rate was 25% in the advanced stage of Barcelona clinic liver cancer staging system [23], and the reported median survival time of extrahepatic spread is approximately 8 months [1, 24]. Ovarian metastases tend to be detected after they become large and exhibit peritoneal involvement, as shown in Table 2, and regular pelvic examination might be essential in patients with HCC, especially after RFA and PEIT. In the present case, it could be retrospectively assumed that the ovarian metastasis emerged in the duration between the second RFA and stereotactic body radiotherapy, when sufficient investigation of the pelvic cavity was not performed. Continuous increase of AFP after the second RFA might reflect the ovarian metastasis. Actually, AFP reflected progression of peritoneal dissemination and intrahepatic lesions after the salpingo-oophorectomy. Several studies reported that radical or palliative surgery of extrahepatic sites could significantly prolong survival in patients with good tumor control in the liver and other organs [3, 4, 12, 25]. Some studies described that surgical resection of peritoneal metastasis could also prolong survival in cases with selected conditions, such as small number of peritoneal nodules, AFP level of less than 200 ng/mL, Child–Pugh A, and well-controlled intrahepatic lesion [26–28]. The median survival time of the reported cases of HCC with ovarian metastasis is 12 months (range 4–54), whereas longer survival times beyond 12 months are expected in patients with previously visible HCC lesions across the body that disappear after surgery.

The present case has survived for 11 years after bilateral salpingo-oophorectomy. We propose that three major factors contributed to the prolonged survival with improved quality of life in the present case. First, surgical resection of the large ovarian tumor might have significantly reduced the HCC bulk in the body. Second, effectiveness of 5-fluorouracil-based regimens to peritoneal nodules might have contributed to suppress tumor progression in our case. The remnant lesions after the salpingo-oophorectomy were located at the peritoneum but not in the liver. Targeting the peritoneal nodules, sorafenib was selected as the first treatment, which was the only evidence-based therapy in those days. The SHARP trial reported that sorafenib was effective in prolonging median survival and time to progression in patients with unresectable advanced HCC [29]. Therefore, it was decided that the present case received sorafenib after recovery from surgical wound as the first chemotherapy. Unfortunately, the present case did not have tolerability to sorafenib, and the duration of administration was only 12 days. The 5-fulorouracil-based regimens were administered from the venous route as an alternative therapy, according to the previous reports [30–32] that described the efficacy of intra-arterial 5-fulorouracil-based regimens with cisplatin or interferon α. Monotherapy of S-1 was also tried, based on the previous report [33]. Currently, it was known that S-1 did not statistically improve overall survival in patients with at least sorafenib-refractory HCC [34].

Finally, hepatic functional reserve could be maintained during multidisciplinary therapy. The significance of hepatic functional reserve in achieving prolonged survival has been frequently reported in patients undergoing surgical resection [27, 35, 36], locoregional therapy [37–39], and molecular-targeted therapy [40–43]. In the present case, balloon angioplasty performed for Budd–Chiari syndrome stopped continuous progression of liver fibrosis and prevented the reduction of hepatic functional reserve. In addition, successful suppression of the intrahepatic lesions by 5-fluorouracil-based regimens aided in minimizing the number of TACE sessions. Low-dose FP was administered approximately three courses per year. As a result, sufficient periods for recovery from adverse effect were obtained and hepatic functional reserve was retained. Ramucirumab also contributed to the suppression of tumor progression without reduction of hepatic functional reserve. In the REACH-2 trial, ramucirumab was associated with improved overall survival compared with placebo [44]. Although the objective response was extremely low, there is a notable case report that continuous ramucirumab treatment for ten months led to partial response with a normal AFP level [45]. Regarding treatment-related adverse effects, good tolerance even in elderly patients was also reported with improvement of overall survival and quality of life [46]. In a network meta-analysis, Wang et al. found that the efficacy of ramucirumab was comparable to regorafenib and cabozantinib in patients with AFP levels above 400 ng/mL, although the adverse effects were fewer with ramucirumab compared to regorafenib and cabozantinib [47]. In the present case, ramucirumab did not decrease tumor volume but dramatically reduced AFP level, and the tolerance was better for ramucirumab than for sorafenib and lenvatinib.

Patients with HCC metastasis to the ovary are usually diagnosed with large-sized tumors with peritoneal involvement; therefore, regular surveillance of the pelvis should be considered especially after hepatic resection, tumor puncture, and HCC rupture. Even in patients with ovarian and peritoneal metastases, multidisciplinary treatment with maintenance of hepatic functional reserve has a possibility of improvement in survival.

In conclusion, multidisciplinary therapy has achieved long-term survival of 11 years with improved quality of life and good performance status in a patient with rare HCC metastasis to the ovary and peritoneum due to favorable maintenance of hepatic functional reserve.
